# Roles and Clinical Applications of Exosomes in Cardiovascular Disease

**DOI:** 10.1155/2020/5424281

**Published:** 2020-06-10

**Authors:** Dong Guo, Yuerong Xu, Jian Ding, Jiaying Dong, Ning Jia, Yan Li, Mingming Zhang

**Affiliations:** ^1^Department of Cardiology, Tangdu Hospital, The Fourth Military Medical University, Xi'an, Shaanxi 710038, China; ^2^Department of Orthodontics, School of Stomatology, The Fourth Military Medical University, Xi'an, Shaanxi 710032, China; ^3^Center for Mitochondrial Biology and Medicine, The Key Laboratory of Biomedical Information Engineering of Ministry of Education, School of Life Science and Technology, Xi'an Jiaotong University, Xi'an, Shaanxi 710049, China

## Abstract

Despite substantial improvements in therapeutic strategies, cardiovascular disease (CVD) is still among the leading causes of mortality and morbidity worldwide. Exosomes, extracellular vesicles with a lipid bilayer membrane of endosomal origin, have been the focus of a large body of research in CVD. Exosomes not only serve as carriers for signal molecules responsible for intercellular and interorgan communication underlying CVD pathophysiology but also are bioactive agents which are partly responsible for the therapeutic effect of stem cell therapy of CVD. We here review recent insights gained into the role of exosomes in apoptosis, hypertrophy, angiogenesis, fibrosis, and inflammation in CVD pathophysiology and progression and the application and mechanisms of exosomes as therapeutic agents for CVD.

## 1. Introduction

Cardiovascular disease (CVD), with a global prevalence of 10.6%, imposes a large health and economic burden [1]. Numerous intra- and extracellular factors, biochemical complexes, and vesicles have been found to participate in regulating the pathophysiological progression of CVD, and studies have suggested that exosomes play important roles in it as well. Exosomes are a subgroup of extracellular vesicles surrounded by a lipid bilayer membrane of endosomal origin and ranging in size from 40 to 160 nm (average~100 nm) [2, 3]. The contents of exosomes include lipids, proteins, mitochondrial DNA, mRNAs, and noncoding RNAs, which are constantly changing both in quality and in quantity according to the microenvironment where the parent cells are [4, 5]. Exosomes are important in intercellular and interorgan communication by delivering signal molecules to recipient cells and regulating downstream signal pathways which have been associated with CVD progression [6, 7]. Moreover, exosomes released by progenitor cells are bioactive and are the key mediators of stem cell therapy of CVD, which simultaneously overcome some limitations of stem cell therapy [8–10].

In this review, we outline the role and function of exosomes in CVD pathophysiological progression and focus on their use to repair the cardiac injury.

## 2. Exosomes in Cardiovascular Pathophysiology

**Figure 1:**
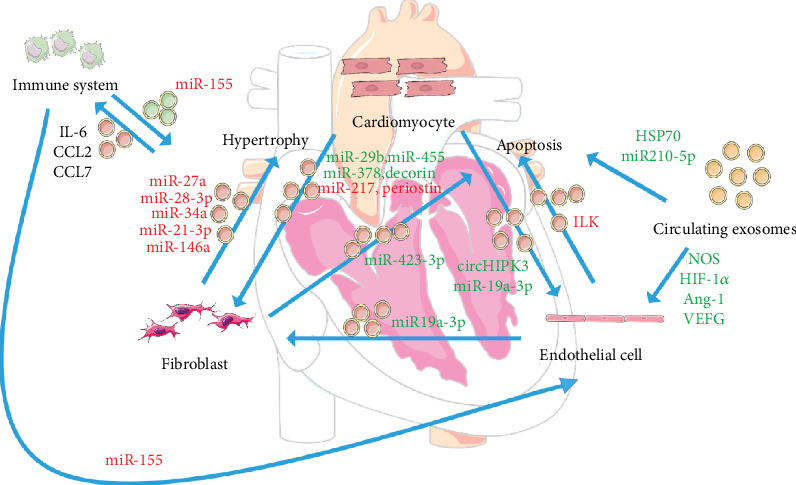
The roles of exosomes in the pathophysiology of cardiovascular diseases. Exosomes serve as the messengers exchanging bioinformation among cardiomyocytes, fibroblasts, endothelial cell, and the immune system. The molecules transported by exosomes regulate the hypertrophy, apoptosis, fibrosis, angiogenesis, and immune response in the recipient cells; those exerting adverse effects are depicted in red, while those depicted in green and black exert beneficial and neutral effects in the corresponding pathological process.

CVD progression entails a series of basic pathological changes, including cardiomyocyte apoptosis and hypertrophic growth, angiogenesis, cardiac fibrosis, and inflammation. Exosomes play a critical role in regulating CVD progression via transport and exchange of signal molecules [11, 12] (see Figure 1).

### 2.1. Apoptosis

The apoptosis of cardiomyocytes is a critical event in CVD progression, including myocardial infarction (MI) and heart failure. By transporting signal molecules between cardiomyocytes and other cells and organs, exosomes regulate the apoptosis of cardiomyocyte apoptosis. For example, exosomes released by cardiac fibroblasts rescue cardiomyocytes from ischemia-reperfusion injury. Mechanistically, miR-423-3p, which is remarkably enhanced in cardiac fibroblasts and exosomes, fulfills its cardioprotective effect by downregulating RAP2C in H9C2 cells and consequently reducing apoptosis [13]. The bioactive factors in the exosomes can also be proteins. For instance, circulating exosomes isolated from healthy volunteers and rats protect cardiomyocytes from apoptosis by the interaction between exosomal HSP70 and TLR4 on the cardiomyocytes and subsequent activation of the TLR4-ERK-p38MAPK-HSP27 pathway [14]. Even the exosomes secreted by cardiomyocyte itself, which is pretreated with lentivirus to overexpress HSP20, can reduce apoptosis via amplifying the AKT signaling pathway [15]. Exosomes also can be negative regulators of apoptosis. In systemic inflammation as mimicked by *il-10* knockout, cardiac endothelial cell-derived exosomes aggravate cell apoptosis post infarction by increasing ILK in exosomes and activating the NF-*κ*B pathway in recipient cells [16]. Moreover, the biological function of exosomes is highly dependent on the status quo of the cell type of origin. While exosomes derived from cardiomyocytes from healthy volunteers significantly promote proliferation and reduce apoptosis of neonatal rat cardiomyocytes in vitro, exosomes from patients with heart failure yield the opposite outcome, which may be secondary to decreased exosomal miR-21-5p which activates PTEN and downregulates Akt phosphorylation in cardiomyocytes. PDCD4, a pro-apoptotic protein, is also a target of miR-21-5p [17].

### 2.2. Hypertrophy

In response to TNF-*α* stimulation, cardiac fibroblasts secrete miR-27a-, miR-28-3p-, and miR-34a-enriched exosomes, which are taken up by cardiomyocytes and suppress the expression of Nfn2 and promote expression of cardiac hypertrophy-related genes such as ANP and *β*-MHC in cardiomyocytes [18]. miR-21-3p-enriched exosomes are also released by cardiac fibroblasts in the mouse model of cardiac hypertrophy, and exosomal miR-21-3p silences SORBS2 and PDLIM5 in cardiomyocytes resulting in cardiac hypertrophy [19]. Exosomes also intensify cardiac hypertrophy by promoting Ang II production and its receptor content in cardiomyocytes [20]. In a heart failure model, miR-146a is overexpressed in active myofibroblasts and secreted through exosomes. Exosomal miR-146a is taken up by cardiomyocyte leading to the dysfunction of SUMOylation [21].

### 2.3. Angiogenesis

The density of myocardial capillary is a critical pathological index of cardiac function and angiogenesis is of great importance in cardiac repair and regeneration post injury [5, 22]. In the MI microenvironment, proinflammatory M1-type macrophages have increased the expression of proinflammatory miRNAs, such as miR-155, and transport them to endothelial cells through exosomes. These proinflammatory exosomes inhibit Sirt1/AMPK*α*2/eNOS and RAC1/PAK2 pathways in endothelial cells which reduces angiogenic potential and aggravate myocardial injury [23]. Besides macrophage, cardiomyocytes in hypoxic environment release exosomes with significantly upregulated circHIPK3, which acts as an endogenous miR-29a sponge, abrogates the inhibition of IGF-1, and relieves oxidative stress-induced dysfunction in cardiac microvascular endothelial cells [24]. Exosomes derived from cardiac telocytes, which is a subgroup of interstitial Cajal-like cells, increase the proliferation, migration, and tube-formation of endothelial cells post MI [25]. Interestingly, a previous study suggests that pretreating the bilateral hindlimbs of rats with tourniquets could enhance the angiogenesis and alleviate the apoptosis of endothelial cells post MI, likely secondary to the proangiogenic contents of circulating exosomes, such as NOS, HIF-1*α*, Ang-1, and VEGF [26]. A novel therapy, low-energy shock wave therapy, also has been proven to enhance cardiac regeneration post MI by improving vascularization. The shock wave therapy can stimulate endothelial cells in the ischemic myocardium to release angiogenic exosomes, and miR-19a-3p is the effective cargo to promote endothelial tube formation and proliferation [27].

### 2.4. Cardiac Fibrosis

Cardiac fibroblasts are indispensable for normal myocardial physiology. However, pathologically activated cardiac fibroblasts deposit excess extracellular matrix, which negatively affects myocardial compliance and stiffness and cardiac function, leading to, for instance, heart failure with preserved ejection fraction [28–30]. Cardiac fibrosis can be modulated by different types of exosomes. Under exercise conditions, diabetic cardiomyocytes release exosomes with higher content of miR-29b and miR-455, which can bind to the 3′ region of MMP9, suppressing its expression and reducing cardiac fibrosis [31]. In the chronic heart failure model, cardiomyocyte-derived miR-217-containing exosomes target PTEN and aggravate cardiac fibrosis both *in vivo* and *in vitro* partly by promoting the proliferation of fibroblasts [32]. Mechanical stress increases the secretion of exosomal miR-378 from cardiomyocytes, and exosomal miR-378 inhibits excessive cardiac fibrosis by suppressing the p38 MAPK-Smad2/3 pathway [33]. Exosomes derived from macrophages are also involved in the pathological activation of cardiac fibroblasts. Exosomes derived from macrophages in a diabetic microenvironment exacerbate cardiac fibrosis, which is abrogated by human antigen R (HuR) knockdown (either in macrophages or in exosomes) [34]. Outsider intervention can also regulate the crosstalk inside a cardiac microenvironment and affect the process of fibrosis. Injection into the ischemic myocardium of exosomes derived from endothelial cells stimulated by shock wave therapy decreases myocardial fibrosis [27]. Simvastatin significantly attenuates collagen deposition and cardiac fibrosis in rats treated with Ang II, which appears secondary to simvastatin-mediated induction of decorin and reduction of periostin in Ang II-treated cardiomyocyte-derived exosomes [35].

### 2.5. Inflammation

The interaction between the immune and cardiovascular systems plays an important and complex role in CVD progression, with exosomes mediating the exchange of signals among cells and organs [36]. Inflammation promotes the secretion of miR-155-enriched exosomes by activated macrophages. Compared with the control group, the myocardium treated with miR-155-enriched exosomes exhibits markedly increased expression of proinflammatory cytokines, including IL-1*β*, IL-6, TNF-*α*, and CCL-2, likely secondary to the suppression of Socs1 [37]. Systematic inflammation is also reported to impair the beneficial effect of endothelial exosomes on cardiac repair post cardiac injury [16]. The “cardioimmune” regulation appears to be reciprocal, as myocardium-derived exosomes can also influence the localization and function of immune cells post cardiac injury. Post MI, cardiac exosomes are rapidly released to the interstitial space but also rapidly disappear, which is reported to be related to immune cell infiltration into the ischemic region. Migrating immune cells, mainly monocytes, engulf cardiac exosomes and increase the expression of IL-6 and chemokines CCL2 and CCL7, which shapes the inflammation post MI [38].

The roles and functions of exosomes in the cardiovascular system have also been characterized in the normal conditions. For example, a study revealed that both in the basal level and in the mild stress state, exosomal HSP60 is continuously released by cardiomyocytes [39]. However, the scarcity of such studies hinders us from figuring out the physiological role of exosomes.

## 3. Application of Exosomes in Cardiovascular Disease Treatment

**Figure 2:**
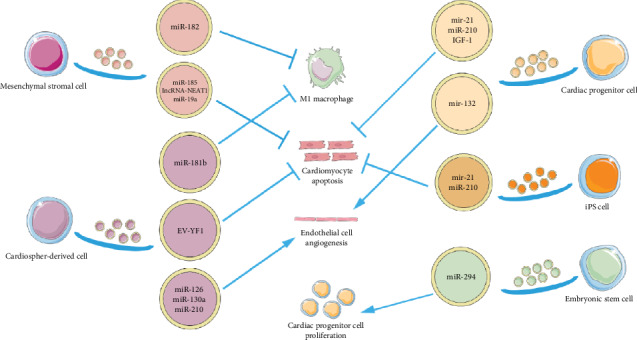
Application of exosomes in the treatment of cardiovascular diseases. Multiple stem cells, namely, mesenchymal stromal cells, cardiosphere-derived cells, cardiac progenitor cells, induced pluripotent stem cells, and embryonic stem cells, secrete exosomes containing therapeutic molecules to modulate the macrophage polarization, cardiomyocyte apoptosis, angiogenesis, and other responses in cardiac injury.

Exosomes are deemed as the bioactive ingredient responsible for the beneficial effects of stem cell therapy in repairing cardiac injury. Exosomes as therapeutic agents can overcome some drawbacks of stem cell therapy, such as the low stem cell retention rate. We here summarize the therapeutic effects of exosomes and their underlying mechanisms (see Figure 2).

### 3.1. Mesenchymal Stromal Cell-Derived Exosomes

Mesenchymal stromal cell- (MSC-) derived exosomes have become attractive candidates for cardiac injury repair. MSC-derived exosomes enhance the polarization of M1 macrophages to M2 macrophages which significantly alleviates the inflammation in the heart and reduces the infarct size. miR-182 knockout in exosomes or TLR4 knockout in macrophages attenuates the immunomodulation function of MSC-derived exosomes [40]. In terms of apoptosis, the content of miR-185 is dramatically increased in exosomes released from MSCs, which suppresses SOCS2 and rescues cardiomyocytes from apoptosis post infarction [41]. MSC-derived exosomal miR-19a also exhibit antiapoptosis effects, by targeting SOX6, activating AKT, and inhibiting JNK3/caspase-3 pathway in cardiomyocytes [42].

Despite the aforementioned salutary effects, the efficacy of exosomes isolated from original and nonartificial MSCs remains limited, which has triggered efforts to optimize and engineer MSCs. Exosomes obtained from MSCs overexpressing MIF show a better cardioprotective effect on reducing mitochondrial fragmentation and cardiomyocyte apoptosis compared to normal MSC-derived exosomes [43]; this is also the case for exosomes secreted by MSCs cultured in a medium containing extra MIF. Mechanically, the exosomal transfer of lncRNA-NEAT1 modulates miR-142-3p and FOXO1 in cardiomyocytes and ameliorates oxidative stress [44]. Interestingly, circulating exosomes isolated from serum post MI can also be a stimulant of MSCs. The serum exosomes, which are proved to mainly come from the kidney and ischemic myocardium, transfer miR-1956 to MSCs, downregulate Notch-1 pathway, and significantly enhance the pancreatic function of MSCs [45]. The efficacy of MSC-derived exosomes is also greatly influenced by the origin of MSCs. For instance, exosomes secreted by human fetal amniotic fluid stem cells (hAFSCs) possess higher cardioprotective potential than exosomes derived from traditional adult MSCs [46].

### 3.2. Cardiosphere-Derived Cell-Derived Exosomes

Exosomes released by cardiosphere-derived cells (CDCs) alleviate cardiac hypertrophy induced by Ang II and reduce myocardial infarct size post infarction. The Y RNA fragment EV-YF1, which is the maximum small RNA inside exosomes, can mimic such cardioprotective effect by altering the IL-10 expression [47, 48]. CDC-derived exosomes alleviate cardiac fibrosis, enhance tube formation of endothelial cells, and attenuate the cardiomyocyte apoptosis post MI [49–51]. Macrophage polarization is also regulated by CDC-derived exosomes after MI. Exosomal miR-181b hinders PKC*δ* expression and enhances M1 to M2 macrophage shift, which underlies the beneficial effect of CDC-derived exosomes [52]. Such a protective effect also has been documented in large animals such as pigs, further underscoring the therapeutic effect of CDC-derived exosomes following intravenous delivery in a myocardial infarction model [53]. Moreover, exosomes obtained from CDCs of neonatal rats or pediatric donors induce cardiac rejuvenation in old ones, as evidenced by the decreased cardiac hypertrophy and myocardial fibrosis, improved cardiac systolic and diastolic function, and enhanced exercise capability [54].

Like MSCs, exosomes obtained from engineered CDCs exhibit an improved protective effect. Hypoxic treatment, as the most widely used preconditioning method, increases the efficacy of CDC-derived exosomes by enriching proangiogenic miRs, such as miR-126, miR-130a, and miR-210, in exosomes [50]. The antiapoptotic effect of CDC-derived exosomes is also reinforced after hypoxic treatment [51]. Preclinical research has revealed that the decreased potency of CDC and its exosomes are associated with the inactivation of Wnt/*β*-catenin pathway, and *β*-catenin can efficiently reverse the therapeutic efficacy of the low potency CDCs [55]. To promote the cardiac tropism of CDC-derived exosomes, CDCs are engineered to connect a cardiomyocyte-specific binding peptide to the N-terminus of Lamp2b, which is a transmembrane protein on the exosomes. This engineered exosomes show better cardiomyocyte-specific uptake and enhanced protective effect [56].

### 3.3. Cardiac Progenitor Cell-Derived Exosomes

Cardiac progenitor cells (CPCs) are also emerging as a promising candidate in stem cell-based therapy for cardiac repair. CPC-derived exosomes effectively attenuate cardiomyocyte apoptosis induced by oxidative stress *in vitro* in an exosomal miR-21-dependent manner, which targets the gene PDCD4 in cardiomyocytes [57]. *In vivo*, reduced apoptosis and improved ejection fraction are observed in the infarcted hearts injected with exosomes released by CPCs, which has been associated with increased miR-210 in exosomes and decreased ephrin A3 and PTP1b in cardiomyocytes [58]. Moreover, tube formation in endothelial cells is promoted by CPC-derived exosomes because the miR-132 in exosomes inhibits the RasGAP-p120 [58]. Exosomes from CPCs perform better in alleviating ischemic cardiac injury than those from bone marrow-derived MSCs. The beneficial effects are associated with the activation of PAPP-A, the release of IGF-1 in exosomes, and the enhancement of intracellular Akt and ERK1/2 [59].

As for expanding the efficacy of CPC-derived exosomes, ticagrelor appears to be an effective stimulant, which can improve both the quantity and quality of CPC-derived exosomes. Ticagrelor enhances the mitotic activity of CPCs and thus increases the number of the exosome level. Meanwhile, exosomes from CPC precondition with ticagrelor show improved antiapoptotic activity through the activation of the ERK1/2 pathway [60].

### 3.4. Other Stem Cell-Derived Exosomes

Exosomes secreted by induced pluripotent stem (iPS) cells protect the heart against multiple stress [61]. iPS cell-derived exosomes enhance the autophagic flux by inhibiting mTOR pathway and promoting cardiomyocyte survival both *in vitro* and *in vivo* [62]. Cardioprotective miR-21 and miR-210 are transported to cardiomyocytes by exosomes obtained from iPS cells, and they suppress caspase 3/7 and improve the function of an ischemic myocardium [63].

Embryonic stem cell-derived exosomes are also effective in relieving cardiac injury [64]. For example, cardiomyocyte pyroptosis induced by doxorubicin can be greatly attenuated by the treatment of embryonic stem cell-derived exosomes, which can decrease the inflammation in the injured myocardium by blocking caspase-1-dependent cell death [65]. Cardiac injury induced by doxorubicin is also ameliorated by the increased number of M2 macrophages and levels of anti-inflammatory cytokines, which is mediated by embryonic stem cell-derived exosomes [66]. Besides promoting neovascularization and cardiomyocyte survival in infarcted hearts, exosomes from embryonic stem cells augment the survival and proliferation of CPCs and promote endogenous repair, which is mediated by the exosomal miR-294 [67].

### 3.5. Nonstem Cell-Derived Exosomes

Cardiac injury also can be alleviated by the exosomes derived from nonstem cells. For example, upon hypoxia stress, cardiac fibroblasts would release miR-423-3p-enriched exosomes and reduce apoptosis of cardiomyocytes [13]. Cardiomyocyte-derived exosomes can reduce the cardiac fibrosis caused by mechanical stress through expanding the content of miR-378 [33]. The response partially belongs to the body's self-repair system, and the targeted activation of the process can be the new treatment strategy of cardiovascular diseases.

### 3.6. Exosome Delivery Method

Exosomes are most commonly delivered via the intravenous route and have been shown to be effective in many animal models and in patients with graft versus host disease [68, 69]. Exosomes also alleviate cardiac injury when administered via an intracoronary route, intramyocardial injection, and cardiac patch [53, 70]. The huge benefits of exosome-based therapy have fueled exploration of more clinically feasible and effective exosome delivery methods, which will greatly promote the clinical applications of exosomes [71, 72].

## 4. Conclusion

Exosomes play critical and important roles in the regulation of physiological and pathophysiological processes, recognition and diagnosis, and treatment of CVD [11]. In this review, we focus on the regulation of pathophysiological process by exosomes and the therapeutic potential, which is of great importance in understanding the mechanism of CVDs and exploring therapeutic approaches. However, there remain many hurdles, including tropism and pharmacokinetics of exosomes, to surmount for their clinical application. Research to further assess the contents of exosomes and the signals exchanged by exosomes will continue, as will efforts to optimize efficacy and delivery methods for potential clinical applications.

## Abbreviations

CVD: Cardiovascular disease
MI: Myocardial infarction
RAP2C: Ras-related protein Rap-2c
ILK: Integrin-linked kinase
NF-*κ*B: Nuclear factor *κ*B
Nfn2: Nuclear factor erythroid 2-related factor 2
SORBS2: Sorbin and SH3 domain-containing protein 2
PDLIM5: PDZ and LIM domain 5
Ang II: Angiotensin II
Sirt1: Sirtuin 1
AMPK: Protein kinase AMP-activated catalytic subunit
eNOS: Endothelial nitric oxide synthase
RAC1: Rac family small GTPase 1
PAK2: p21 (RAC1)-activated kinase 2
MMP9: Matrix Metalloprotease 9
MSCs: Mesenchymal stromal cells
TLR4: Toll-like receptor 4
SOCS2: Suppressor of cytokine signaling 2
MIF: Macrophage migration inhibitory factor
FOXO1: Forkhead class O1
CDCs: Cardiosphere-derived cells
PKC*δ*: Protein kinase C*δ*
CPCs: Cardiac progenitor cells
PDCD4: Programmed cell death 4
PAPP-A: Pregnancy-associated plasma protein-A
IGF-1: Insulin-like growth factor-1
iPS cells: Induced pluripotent stem cells
mTOR: Mammalian target of rapamycin
HSP60: Heat shock protein 60.
